# Exploring curiosity in undergraduate medical education: a thematic analysis

**DOI:** 10.1186/s12909-026-08783-x

**Published:** 2026-02-11

**Authors:** Khui Chiang Wee, James Lavery, Hugh Alberti

**Affiliations:** School of Medicine, Newcastle University, Newcastle Upon Tyne, Framlington Place, Newcastle upon Tyne, UK

**Keywords:** Curiosity, Medical education, Qualitative, Thematic, Undergraduate.

## Abstract

**Background:**

Although curiosity in medicine is associated with many benefits, the literature is generally sparse or anecdotal. We seek to contribute to the understanding of curiosity by exploring its perception in the context of undergraduate medical education.

**Methods:**

Given its subjective and abstract concept, we utilised a qualitative approach in this research. Focus groups and one-to-one semi structured interviews with medical students, clinical teachers and senior curriculum leaders were undertaken. Data collected was thematically analysed using Braun and Clarke model.

**Results:**

All participants felt curiosity was important for learning and patient care. Curiosity was perceived to be a dynamic process – from the initial stage of knowledge acquisition to holistic practice. Teachers were identified as agents to nurture curiosity. Positive role modelling, enthusiasm in teaching and effective teaching styles were some ways to achieve this. Exams were deemed to hinder the development of curiosity. Participants felt curiosity could be nurtured through an ad-hoc basis during students’ day-to-day placement and structured learning activities.

**Conclusions:**

We recommend that medical schools review their existing curriculum to identify more opportunities for curiosity development and eliminate potential barriers. Regular workshops for teachers could raise awareness on nurturing curiosity and to develop effective teaching skills.

**Supplementary Information:**

The online version contains supplementary material available at 10.1186/s12909-026-08783-x.

**Supplementary Information:**

The online version contains supplementary material available at 10.1186/s12909-026-08783-x.

## Background

The practice of curiosity in medicine is associated with reduction in clinical error, improved patient care, burnout prevention, better judgement and memory [1–3], although reports are often sparse, anecdotal and evidence is mostly lacking. One possible attribution is its implicit nature, making it challenging to be explicitly described and quantified [4]. Unlike the practice of observable clinical skills, application of curiosity is dependent on clinicians’ traits such as regular mindfulness, being inquisitive and acquiring skills in capturing patient’s perspectives [5–7]. This indicates curiosity is best nurtured. During workplace learning, students learn through active observation. Social cognition theory highlights that learning is influenced by dynamic interaction between a learner’s traits (self-efficacy, personality, observational learning) and external factors (learning environment, teachers, learning stimuli[8]). Teachers play an important role in this process. They foster a stimulating environment for curiosity learning. For example, setting learning activities that promotes curiosity, role model curiosity application when seeing patients and finding appropriate patients for students to review. Vivekananda-Schmidt et al [9] suggest role modelling is a dynamic process involving experiences and interpretation between students and teachers. With time, curiosity practice is embedded as part of students’ professional identity of a doctor. As professional identity evolves with time [9], a student’s personal definition on curiosity will evolve too.Many factors influence curiosity learning, broadly divided into organisational, cultural and personal influences [10, 11]. Although medical students tend to be high academic achievers at high school or college, this does not necessarily correlate to high levels of curiosity. Medical students come from different backgrounds, such as widening access programmes and international students, meaning that curiosity as a personal trait cannot be presumed. Discussion surrounding curiosity development from the very first stage of a medical career as a student is often neglected. Furthermore, developing learning opportunities to nurture curiosity can be challenging given the vast amount of learning outcomes that are needed to be covered by the existing complex undergraduate medical curriculum [12].Curiosity as a construct is known to be difficult to define [13], though one definition used in the medical education literature is by Gossnickle [14]: ‘the desire for knowledge or information in response to experiencing or seeking out collative variables, accompanied by positive emotions, increased arousal, or exploratory behaviour’ (p.37). As a construct, it can be viewed as a trait or as a reaction to a particular situation. Dyche and Epstein [15] include cognition, socialisation and affective components when describing the idea of curiosity. Fitzgerald [16] suggests the practice of curiosity in medicine stimulates both imagination and intelligence. Schattner [11] associates curiosity with an inherent interest in knowing something observed or someone encountered. These wide-ranging perspectives suggest curiosity is an abstract concept and is considered an important though an underexplored aspect of medical education. A recent paper suggested an epistemic-emotion-framework for understanding and encouraging curiosity but stressed that medical educators need to explore curiosity in various educational contexts [13].

There are attempts to quantify curiosity. Bugaj et al. [17] utilised a scale to measure intellectual and social curiosity among medial students while Sternszus et al. [18] measured trait and state curiosity among medical students using a different inventory. Although both these studies suggest a change in pattern of curiosity measured through the years of the medical programme, a direct comparison of the studies is not possible due to different elements of curiosity measured. It is also not known which components of the curriculum have influenced the change in curiosity. Conversely, Vossler et al. [19] applied Kashdan’s Five-Dimensional Curiosity Scale to medical students to determine if curiosity changes across medical school, finding domain averages were not significantly different across years of medical school.

In this study, we therefore sought to expand the literature surrounding curiosity in the undergraduate medical education setting by conducting focus groups with medical students and clinical teachers, and one-to-one interviews with senior management staff. We explored their perspectives on the concept of curiosity with the following research questions:

- What is curiosity in medical education?
- What are the perceived factors that encourage and hinder the development of curiosity?
- How can curiosity be nurtured?

## Methods

Curiosity is abstract: participants give individualised meaning towards this concept through personal interpretation and experience. We therefore explored our research questions through an interpretivist paradigm (how participants interpret and make sense of meaning within their world), with a relativist ontology (multiple realities exist shaped by participants’ perception) and subjective epistemological lens (participants impose meaning on a subject). We used a qualitative explorative approach for this research and therefore due to confounding factors, as detailed below, we aimed for transferability (the extent to which the research findings can be applied to other contexts or settings) rather than generalisability (pertaining to the ability to apply findings to the larger population.)

Data collection was carried out nearing the end of university summer term as it was the least busy period for participants. We utilised a targeted convenience sampling approach in recruiting participants [20]. We sought to ascertain the views of the students themselves who are experiencing undergraduate medical education, teachers who are directly involved with students and members of the senior management team (SMT) who manage and implement the curriculum. Potential participants were invited to participate via email. Year four students undertaking a Student Selective Component (SSC) in medical education were selected as they had completed the majority of their training, and given their interest in medical education, were expected to have views on curiosity learning. Clinical teachers consisted of General Practitioner (GP) teaching fellows who work at the medical school and were invited due to their interest in this topic. SMT leaders included faculty clinicians with senior influential roles within the medical school and were selected due to their teaching experience and their position to influence teaching policy in the medical school. The mix of students, teachers and SMTs gave a broad mix of viewpoints, from a learning perspective to teaching and policy setting. Students and teachers participated in separate focus groups to encourage contribution without apprehension given they might recognise each other. Due to availability reasons, SMTs had one-to-one interviews.

Fourteen students, ten teachers and five SMTs were invited. Of these, nine students, five teachers and all SMTs participated. Students were split into two focus groups (KW and JL conducted one each) and teachers were in one focus group (conducted by KW). JL conducted the one-to-one interviews with SMTs. These were done either face-to-face at the university (audio recorded) or virtually (visually recorded). All focus groups and interviews used a semi-structured framework with questions developed from the literature and the research team’s experience (appendix 1). Interviews were recorded and transcribed verbatim. Although KW and HA have teaching roles, they had never been involved in teaching the recruited student participants. We acknowledged potential influence between KW and teacher participants, JL and student participants. The decision for KW to interview teachers and JL to interview students was to encourage openness in conversation. It was felt that more honest and accurate results would be gained by interviewed by a peer or near peer. JL interviewed SMTs due to more flexibility in their schedule. Both researchers reassured participants of their impartiality during focus groups and recorded their feelings at the end for reflection.

**Fig. 1:**
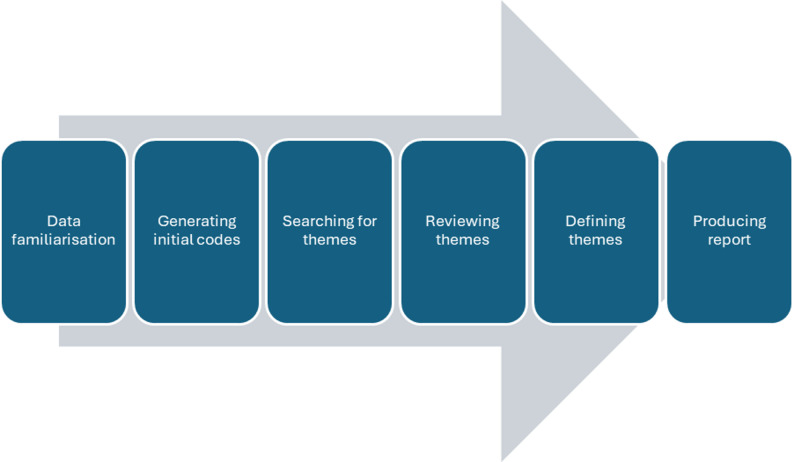
the process of thematic analysis via Braun and Clarke model [21]

Data collected was analysed via thematic analysis using the Braun and Clarke model [21] (Fig. 1). KW and JL participated in this process separately. Transcripts were read multiple times to allow deep immersion, followed by coding and generation of themes using Nvivo software following a bottom-up inductive process. Thematic saturation was used to assess data adequacy. KW and JL exchanged findings generated before mutually agreeing on themes. KW and JL acknowledged how their relatively novice experience in research might influence their observations and interpretation of transcripts. Furthermore, KW and JL were at different phases of their medical careers. KW was a GP trainee at the point of research – he recognised how his experience as a student and then a resident doctor in a GP speciality might influence his personal definition of curiosity. JL was a student at the point of research – he recognised how his perspective on curiosity might be ‘premature’ given his early phase of career. Both acknowledged the impact of their backgrounds on the thematic analysis process, particularly coding and reviewing themes. They reflected after each focus group or interview and were supervised by HA, who acted as verifier of data analysis. HA is an experienced researcher and professor of general practice education. Draft findings were presented to participants to confirm data accuracy. Interaction between participants and researchers stimulated discussion which contributed towards thematic development. This step increased data credibility and also prevented KW’s and JL’s personal perspective on curiosity from influencing data analysis. We have attached COREQ checklist to demonstrate rigor [22] (supplementary material).

## Results

**Fig. 2:**
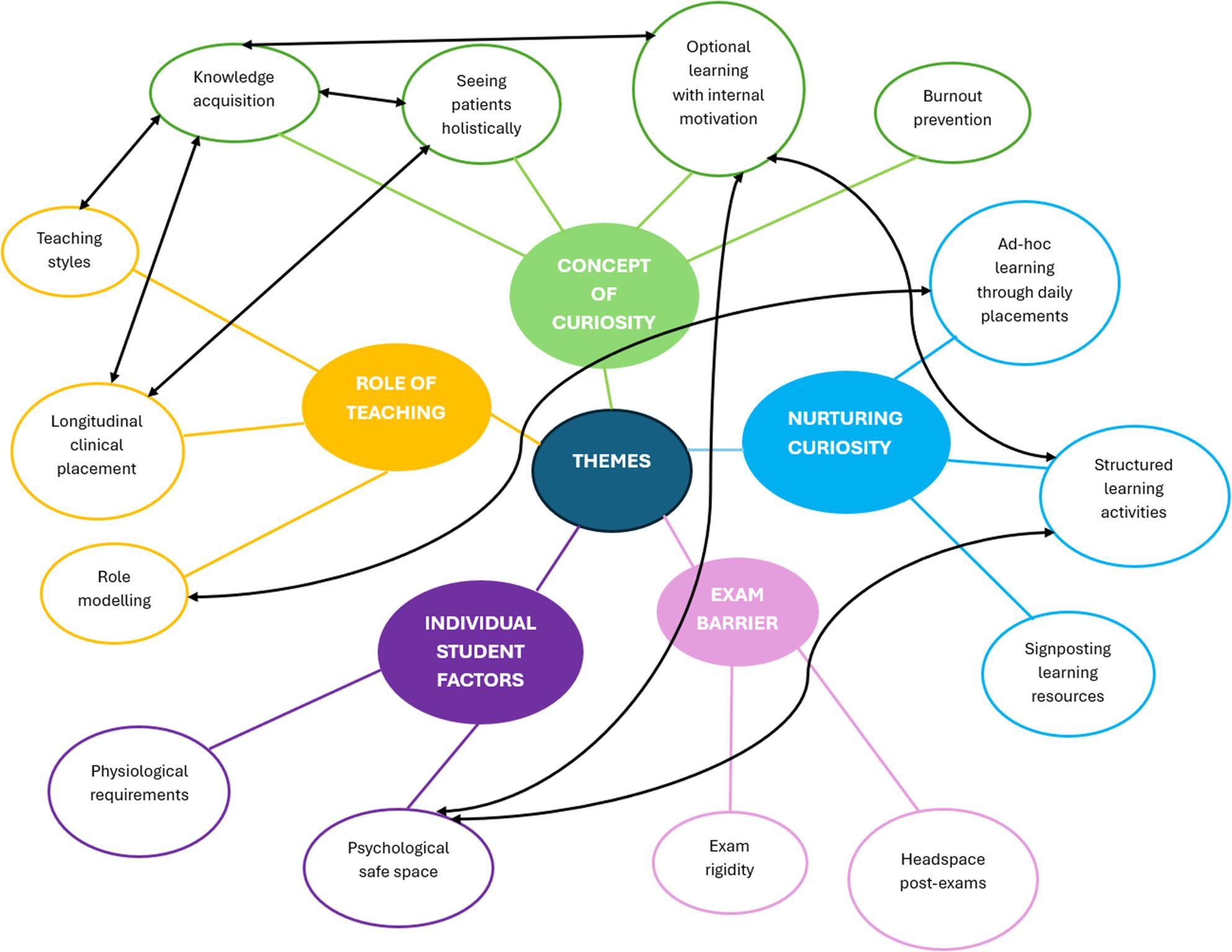
Thematic map. Themes are represented by different colours, with sub-categories in each theme representing a code. Interrelationships between codes are represented by double-sided black arrows

Five themes were generated (Fig. 2). Focus groups lasted between 60 and 90 min and the interviews between 30 and 45 min. Quotes from student focus groups are labelled as SFG1 and SFG2 respectively, clinical teacher focus group as CTFG and SMTs were individually numbered.

## Concept of curiosity

Participants viewed curiosity from two interlinked perspectives.

### Knowledge acquisition

From a student’s perspective, they reported curiosity as a self-motivated desire to seek improvement through knowledge acquisition.

*“Curiosity is important*,* so you go out of your way to learn new stuff and keep up to date*,*” - SFG1*.

*“Sometimes it can be hard to be curious when you know nothing about what you’re doing. I knew nothing about breast cancer stuff (during a clinic placement) until we got to CDM (tutorial) week” – SFG2*.

### Seeing patients holistically

From a clinician’s perspective, being curious meant assessing patient’s presentation as a whole, rather than solely focusing on the clinical aspect of a presenting issue.

*“I think it means having the time*,* space and opportunity to be creative*,* and think about what holistic options are*,* rather than learning from a textbook or from a curriculum on what is needed to know” – CTFG*.

Participants reported curiosity practice allowed them to discover a more in-depth view of a patient’s life, which allowed them to form a more holistic management plan for them. All participants felt that curiosity was important for improved patient care although there was an acknowledgement that it could not be objectively measured. Rather, it relied on students’ or clinicians’ lived experience when seeing their patients.

*“That realisation about changing your viewpoint or seeing the problem differently*,* then that opens up to you a whole lot of different avenue of inquiry and the different area that you’re interested in exploring. And there’s different questions and paying attention to different things.” – SFG2*.

### Optional learning with internal motivation

Students reported curiosity enabled them to identify learning gaps which encouraged them to do further reading. This facilitated more meaningful learning, leading to knowledge acquisition. A common point elicited was that curiosity involved learning beyond the minimum level required by the curriculum. Such learning required personal time and self-motivation.

*“There’s always opportunity for further reading. if I was curious about a topic I guess I’ll maybe go and read further” – SFG1*.

The idea of doing extra reading was met with resistance by some student participants if it was done outside of regular study time; learning through curiosity was perceived to be an optional activity.

Reflecting back to their personal undergraduate training, clinical teachers felt they paid little attention to curiosity learning. They wondered if uninterested students see it as a gimmick, suggesting the concept of curiosity learning as a self-motivated aspiration.

### Burnout prevention

All participants believed being curious prevents burnout in the long term. Burnout increased hurried clinical judgement, thus suppressing curiousity and vice versa. SMTs felt curiosity might impact students’ careers in the future, both in terms of specialty choice and career satisfaction.

*“I’m sometimes a little bit worried and conscious of the fact that when there’s time pressure (during clinic)*,* you become less curious” - SMT4*.

## Role of teaching

### Role modelling

The approach to delivering teaching was cited as one of the most important drivers of curiosity. Role modelling was seen as crucial, with both students and teachers agreeing on the importance of teaching in a certain way, such as providing a ‘safe space’ for students to learn and not castigating students for incorrect answers. Time for reflection and debrief post clinical encounters were mentioned as useful for exploring topics in more detail.

*“Whether it’s role modelling behaviour*,* whether it’s good teaching*,* teaching behaviour or whatever*,* it’s got to be a safe relationship. So it’s safe space*,* safe time*,* safe relationship I think would be the three things that I would put out there as a priority.” – SMT2*.

### Teaching styles

The style of teaching was viewed as crucially important by students. Teachers had to be enthusiastic over the content being taught with an opportunity for freedom of learning. This fed into the concept of a ‘stimulating curriculum’ with students preferring to be cognitively stretched when dealing with uncertainty.

*“I know from personal experience the thought-provoking lectures and clinical teaching for me are ones that give sound background knowledge but then leave some questions unanswered that make me want to go and do more about it” – SFG2*.

Compared to ‘old school’ didactic teaching, non-student participants reported a preference for structured learning when in a clinical environment such as assessing patients and then reporting findings during ward rounds. It was felt such placements also gave time for relationships between students, staff and patients to flourish.

*“Getting students to develop relationships with the clinical teams and patients*,* I think promotes curiosity. That’s what we’re trying to do… get them to follow up the panel patients they have” - SMT5*.

### Longitudinal clinical placement

Longitudinal placements received a mixed response. Facilitators felt embedding a student in a team for an entire year was a good opportunity to build relationships and explore an area of practice to a greater extent. Concerns were noted by students about being placed in an area of medicine too complex for their degree.

Clinical placements gave the opportunity for students to talk and understand more about patients and their conditions. Such situations were felt to help give context to conditions and ‘bring it to life’ more than reading them in a textbook. Interest in patients helped drive curiosity in students and help learning.

*“When you see a patient with a certain condition*,* you go and learn about it. It’s the interest or curiosity that drives you to go and find out about it” - SFG1*.

## Individual student factors

### Physiological requirements

Physiological requirements were noted by SMTs and students as important as per Maslow’s hierarchy of needs [23]. Curiosity was felt to be top of the pyramid as part of self-actualisation and therefore reliant on physiological requirements being met.

*“So people are not going to be curious if they are scared*,* they’re not going to be curious if they’re*,* you know*,* freezing cold or got a little bladder or been on a ward for five hours or something” – SMT1*.

### Psychological safe space

Perceptions of other students during classroom teaching was felt to be important.

*“When you’re in a classroom of 30 people*,* you don’t really want to ask a million questions in front of those 30 people because you’re like*,* oh*,* I don’t want to be that kid in class.” – SFG2*.

This was linked to the idea of small group teaching and optional sessions being preferable by both students and facilitators. Peers in these sessions would also have the same levels of curiosity. Such individual interests were mentioned as a key driver of curiosity and therefore, the option to explore these were felt as beneficial. Motivation to learn more about specific conditions as per their interests helped foster curiosity and included a personal desire to acquire more knowledge. Such a drive to learn more about aspects of medicine included a desire from students to help improve patient care and outcomes.

## Exam barrier

### Exam rigidity

Exams were reported by students as a hindrance towards the development of curiosity. Time and focus were spent on revising for exams and thus students side-lined their interests and curiosity in pursuing learning activities that were not part of the assessment. This thought was echoed by clinical teachers’ past experience.

*“I think that in medical school my memory is just like desperately clinging on to try and pass exams… And so if it’s like*,* Oh*,* that’d be nice to know*,* because it’s interesting*,* it’s like it’s gone.” – CTFG*.

### Headspace post-exams

Both student and teacher participants described a change in mindset towards learning post-exams, with more emphasis on areas that sparked their curiosity. This was attributed to having the time and headspace to explore areas of interest but not necessarily required by exams. Teachers did not feel that curiosity can or should be assessed formally. They felt assessing curiosity removed its very nature and uniqueness.

## Nurturing curiosity

### Ad-hoc learning through daily placements

All participants felt that curiosity learning should be nurtured throughout the undergraduate training. This can be achieved via ad-hoc learning through students’ day-to-day placement. For example, creating an environment that encourages curiosity during patient encounters and reflect on this during debrief opportunities. This reinforces the effect of role modelling as mentioned above.

*“I share that with people quite regularly*,* and talk to them about how it’s helped me and how it’s changed things*,* and I guess I’m always asking why*,* and encouraging them to ask why*,* and seek out extra learning opportunities. I hope my continued encouragement encourages them to do it.” – CTFG*.

### Structured learning activities

Curiosity development was thought to be achievable by all through structured learning activities such as SSCs and electives. Unlike core placements, these placements provided students the freedom to explore an area of their interest. This could promote self-motivation which in turn, encouraged curiosity learning.

*“Students pick (an SSC) which they want to be there*,* so they are curious about that career option. I love talking to my year fives – what did you do in your SSC? In your elective? What did you learn?” - SMT3*.

Clinical teachers felt the timing of these placements should be carefully planned such as after the exam period. This removed stress and provided students the autonomy to pursue learning they were interested in.

### Signposting learning resources

Student participants suggested changes to existing learning resources. They felt an effective learning resource should provide clear signposting between mandatory and optional learning. Such an approach could increase time efficiency as free time could be used to develop curiosity through optional learning, assuming students were interested in them.

*“I think they (learning resources) need to be more succinct. This is the bare minimum of what you need to know and with the rest*,* people can choose to engage with it or not for their interest.” - SFG1*.

## Discussion

Our study indicates that nurturing curiosity in undergraduate medical education is a complex process, yet crucial for the study and practice of medicine. Curiosity is perceived to be a spectrum, from knowledge acquisition at the initial stage of undergraduate medicine to transitioning towards holistic practice during the postgraduate stage; student participants focused on knowledge acquisition whereas teacher participants focused on viewing patients holistically. Epistemic curiosity is known to drive knowledge seeking which results in deeper level learning [24, 25]. We propose that once a sufficient knowledge base is formed, headspace is created for social and affective curiosity when seeing patients [17]. This indicates the dynamic evolution of curiosity with time as students develop their professional identity as a doctor [9]. The experience is personal as they transition at different points of their career journey, influenced by different learning experiences. However, this relies on the assumption that the student feel curiosity practice should form part of their professional identity. Although existing literature describe the different types of curiosity [15, 16, 26], no previous studies have elicited this transitioning of curiosity traits through the stages of medical training.

Many factors contribute to the nurturing of curiosity, widely divided into personal intrinsic and external factors [11, 27]. Our study highlights that these factors are intertwined and interdependent. For example, effective teaching strategies and role modelling of curiosity spark internal motivation in students to pursue further optional reading. The interdependent relationship between these various factors affirms the complex nature of curiosity practice [28].

The role of the teacher and teaching style are key themes noted to influence the nurturing of curiosity. Role modelling, curiosity questioning and small group structured learning contribute to effective teaching strategies [29, 30]. These feed into a stimulating curriculum. Approaches such as appreciating medical complexity, promoting uncertainty with appropriate debriefing and active reflection, achieved through regular patient contact, stimulate curiosity [10, 31]. Furthermore, appropriate questioning styles to help students who are near to bridging their knowledge gap can promote curiosity learning [32]. Small group learning provides a comfortable space for students to ask and answer questions more freely. In fact, Schwarz et al. [13] suggest incorporating elements of novelty, surprise and uncertainty during teaching to stimulate curiosity. This reaffirms the multi-dimensional interaction between teachers, students and patients which contribute to students’ curiosity development, as proposed by the social cognition theory [8]. Such interaction is non-linear and each student attaches different meaning to their interaction due to varying learner’s traits. For example, one may be motivated to reflect on the effect of empathy expression while the other may learn stock-phrases for exams, following debrief from a patient consultation. The difference in students’ ‘take home’ message should not be controlled or viewed negatively; rather teachers should acknowledge the differences and provide appropriate guidance for them throughout their learning.

Students’ curiosity thrives in a learning environment that is physiologically and psychologically safe. This is akin to the higher stages of Maslow’s hierarchy of needs such as self-esteem and self-actualisation [23]. Basic requirements such as regular comfort breaks, small group learning and optimum amount of teaching content need to be met first. Psychological safety can be seen in two aspects. From day-to-day learning, teachers should deliver learning content in a way that encourages students to feel safe to ask questions. This allows them to explore their thoughts and perk curiosity. From a curriculum perspective, planners should create formal learning opportunities for students to explore an area of their interest. Examples include SSC and electives. Providing them an autonomy to pursue further learning of their choice reflects on internal motivation, thus promotes curiosity learning [33]. However, integrating such placement is challenging given the vast amount of mandatory learning and assessment imposed by external regulatory bodies.

Exams prevent students from achieving higher stages of the Maslow’s pyramid due to their stress-inducing nature [33 , 34] and may be a causal factor of ‘ethical erosion’. By focusing on exams, students lack headspace for curiosity practice. For example, they may explore a patient’s concerns because it will be assessed, without considering its impact on subsequent management plan. Yet medical institutions continue to focus on formal, high-stake assessments. Some believe summative assessments drive learning due to extrinsic motivation [35] whereas others believe formative assessments promote intrinsic motivation, improve knowledge acquisition and avoid superficial learning [36–38]. Our findings suggest curiosity is an intrinsic, long-term motivator. Nurturing curiosity learning feeds into higher knowledge acquisition which logically, should result in higher attainment in summative assessments. We propose higher emphasis in incorporating formative assessments which are generally low-stake and less stress-inducing as part of programmatic assessments, to minimise the negative aspects of summative exams. Such an emphasis on small group teaching and formative assessments may help to mitigate institutional barriers to curiosity [15] seen in hierarchical teaching formats and allow more time in the curriculum for self-reflection. An example of ‘non-conventional’ formative assessment would be to prioritise quality improvement projects of the student’s choice.

The practice of curiosity is linked to prevention of burnout in our study. Curiosity works in two directions – outward curiosity towards a patient presentation and an inner curiosity within the mind [39]. The former stimulates cognition whereas the latter stimulates mindfulness. Previous studies have suggested that being overly curious among physicians can lead to burnout due to excessive effort for discovery [27] but our participants did not report any negative effects associated with curiosity. We wonder if the difference may be due to the predominance of general practitioners and trainees in our study although there is no existing literature, to our knowledge, that investigates a comparison between curiosity and different medical specialties. However, we postulate that the nature of general practice which deals with medical complexities and managing uncertainties, whilst mindfully consulting with patients [40, 41], may have contributed to the positive perception towards curiosity. Other benefits to curiosity have been previously explored. Fitzgerald for example [16] postulates that curiosity aids empathy development when practicing medicine and interacting with patients.

There has been previous examples of trying to measure such curiosity in medical students. A 2004 study by Vossler et al. [19] applied Kashdans five dimensional curiosity scale to curiosity in medical students. We also found curiosity to be motivated by similar factors. Themes linked to Joyous Exploration (preference for new expansion and experiences, valuing self explanation over security) were commonly mentioned by students, with self-motivation to expand knowledge. Social Curiosity (interest in acquiring information on how other people feel, think and behave) was more evident in clinicians with a perceived base level of knowledge compared to students. Deprivation Sensitivity (seeking information to escape the tension of not knowing something) was mentioned by less, however students noted concern that not all medical conditions required to be known for practice would be supplied in teaching.

Our research demonstrates the importance of nurturing curiosity in undergraduate medical education. Curriculum planners should emphasise small group teaching and incorporate learning activities which promote autonomous learning. These should motivate students to acquire knowledge which they can apply when seeing patients during placement. However, overhauling the curriculum can be challenging given its vast amount of existing interlinked content to be covered. Role modelling curiosity during patient encounters and creating a safe space for students to reflect, are some effective strategies that teachers can consider to nurture curiosity. This largely depends on the teachers’ interest. Workshops are key to raise awareness and encourage teachers to exchange ideas on nurturing curiosity. From our data collection, teachers felt curiosity should not be formally assessed, which is in line with its abstract nature. This means any targeted intervention to promote curiosity cannot be directly measured. However, we hope these workshops guide teachers on how to informally evaluate students’ curiosity level during learning activities. For examples, engagement levels with teaching activities and creating space for curiosity questioning. Workshops have not been held as a result of this study and this is currently a limitation to our study. Our next research should focus on the design of these workshops and evaluate their impact in equipping teachers with the necessary skills to nurture curiosity. We recommend further research into evaluating ways to increase curiosity from the perspective of medical students, whether this be by course design or targeted extracurricular activities. Larger studies evaluating curiosity opinion by university year would be beneficial.

Previous studies have examined curiosity in medical education, however the literature is still sparse in this area. To our knowledge, our research is one of the first to explore the perception of curiosity learning with participants across different journeys of their medical careers. Linked is the attempt to marry the opinions of people at different career stages to suggest improvements to education practice. Although our targeted convenience sampling approach limits generalisability, we believe the findings may be transferable with further studies in other settings. As our student participants were selected from the clinical years, further exploration could be considered amongst pre-clinical undergraduates.

In terms of methodological rigour, we aimed to follow Lincoln and Guba’s Framework of credibility, dependability, confirmability and transferability [42]. These were achieved through our reflexive, iterative and team approach, immersion in the data, a clear analytical audit trail and by presenting initial findings to our participants, We acknowledge our small sample size and teacher participants who were mostly from one specialty background may affect transferability. Research with a larger sample size, from different institutions and wider participation from different specialties is warranted. The research was conducted at the end of the Summer term and into the holiday period. We acknowledge that student perceptions of curiosity may differ in this time period as it is post exams and in a ‘low intensity’ period, compared to earlier in the academic year. Examining curiosity and any change in perceived importance to students throughout the year would further aid in examining curiosity as a dynamic process.

## Conclusion

We have explored curiosity in the context of undergraduate medical education. Our research proposes that curiosity learning and its application is a transitional spectrum with many interplaying factors. Educational stakeholders should integrate opportunities for curiosity learning through a multi-level corporation approach. Further research is needed to explore the gaps identified in this research.

## Supplementary Material

Supplementary Material 1.

## Data Availability

The datasets analysed during the current study are available in an anonymised format from the corresponding author upon reasonable request.

## Appendix

Appendix 1 is a word format document entitled ‘Interview schedule.’ It shows the introductory statement prior to each interview or focus group, before showing the different interview sections and questions used to guide the interview.

## Introduction

Thank you for offering your time to participate. I hope you have had the opportunity to read the participant information sheet and sign the accompanying consent form. If you have not done so and still wish to participate I would encourage you to do so now. I will echo these documents in saying that this interview is being audio-recorded, but your responses will remain anonymous, and the recording kept confidential.

The intention of this interview is to explore the perception of learning/teaching of curiosity in the undergraduate medical degree at Newcastle University.

### Introductory questions

1. What does curiosity mean to you?
2. Have you ever thought about curiosity in your training and whether it is important?
3. Do you think it might be important for medical students and doctors? If so, why?

### Section A: perceived factors in the development of curiosity at medical school

4. What are the factors you feel that encourage the development of curiosity during the undergraduate medical course?
5. What are the factors you feel that hinder the development of curiosity during the undergraduate medical course?
6. Any thoughts on how teachers or tutors, may have contributed to the encouragement and hindrance of the development of curiosity

## Use prompts if needed

### Section B: teaching or nurturing of curiosity development at medical school

How do you think curiosity could be taught or nurtured at medical school? Think about the role of students, teachers, curriculum or anything else that you feel important to you.

### In conclusion:

- Anything else to add from our discussion above?

## Abbreviations

GP: General Practitioner
SSC: Student selective component
SFG: Student focus group
SMT: Senior management team
CTFG: Clinical teacher focus group
KW: Khui Wee
JL: James Lavery
HA: Hugh Alberti

## Glossary

Student Selective Component: – A 4-week module block where medical students are free to sign up to a placement, clinical or non-clinical, of their own choice.

## References

[1] Krasner MS, Epstein RM, Beckman H, Suchman AL, Chapman B, Mooney C, et al. Association of an educational program in mindful communication with burnout, empathy, and attitudes among primary care physicians. JAMA. 2009;302(12):1284–93. 19773563 10.1001/jama.2009.1384

[2] Redelmeier DA. Improving patient care. The cognitive psychology of missed diagnoses. Ann Intern Med. 2005;142(2):115–20. 15657159 10.7326/0003-4819-142-2-200501180-00010

[3] McGillivray S, Murayama K, Castel AD. Thirst for knowledge: the effects of curiosity and interest on memory in younger and older adults. Psychol Aging. 2015;30(4):835–41. 10.1037/a0039801. 26479013 10.1037/a0039801 PMC4679590

[4] Adashi EY, Ahmed A-KH, Gruppuso PA. The importance of being curious. Am J Med. 2019;132(6):673–74. 30571933 10.1016/j.amjmed.2018.12.002

[5] Derksen F, Bensing J, Lagro-Janssen A. Effectiveness of empathy in general practice: a systematic review. Br J Gen Pract. 2013;63(606):e76–84. 10.3399/bjgp13X660814. 23336477 10.3399/bjgp13X660814 PMC3529296

[6] Epstein RM. Mindful practice. JAMA. 1999;282(9):833–9. 10478689 10.1001/jama.282.9.833

[7] Levinson W, Gorawara-Bhat R, Lamb J. A study of patient clues and physician responses in primary care and surgical settings. JAMA. 2000;284(8):1021–7. 10944650 10.1001/jama.284.8.1021

[8] Bandura A. Social foundations of thought and theory: A social cognitive theory. Englewood Cliffs, NJ: Prentice-Hall; 1986.

[9] Vivekananda-Schmidt P, Crossley J, Murdoch-Eaton D. A model of professional self-identity formation in student Doctors and dentists: a mixed method study. BMC Med Educ. 2015;15(83). 10.1186/s12909-015-0365-7. 10.1186/s12909-015-0365-7 PMC4422527 25924676

[10] Ince-Cushman DJ, Dove M, Jarvis C, Opatowski D. Curiosity as a tool in medical supervision. Can Fam Physician. 2022;68(10):783–4. 36241394 10.46747/cfp.6810783 PMC9833134

[11] Schattner A, Curiosity. Are you curious enough to read on? J R Soc Med. 2015;108(5):160–4. 26022549 10.1177/0141076815585057 PMC4484215

[12] Kopelman P. The future of UK medical education curriculum - what type of medical graduates do we need? Future Hosp J. 2014;1(1):41–6. 31098043 10.7861/futurehosp.14.011 PMC6438223

[13] Schwarz TA, Nikendei C, Cranz A, Friederich H-C, Bugaj TJ. An untapped potential: curiosity in medical school. Med Teach. 2024;46(7):939–47. 38048416 10.1080/0142159X.2023.2288546

[14] Grossnickle EM. Disentangling curiosity: dimensionality, definitions, and distinctions from interest in educational contexts. Educ Psychol Rev. 2016;28(1):23–60.

[15] Dyche L, Epstein RM. Curiosity and medical education. Med Educ. 2011;45(7):663–8. 21649698 10.1111/j.1365-2923.2011.03944.x

[16] Fitzgerald FT, Curiosity. Ann Intern Med. 1999;130(1):70–2. 9890857 10.7326/0003-4819-130-1-199901050-00015

[17] Bugaj TJ, Schwarz TA, Terhoeven V, Nagy E, Cranz A, Friederich H-C, et al. Measuring an understudied factor in medical education – development and validation of the medical curiosity scale. Med Educ Online. 2023;28(1):2198117. 37014965 10.1080/10872981.2023.2198117 PMC10075518

[18] Sternszus R, Saroyan A, Steinert Y. Describing medical student curiosity across a four year curriculum: an exploratory study. Med Teach. 2017;39(4):377–82. 28379089 10.1080/0142159X.2017.1290793

[19] Vossler K, Tan R, Christensen W, Lockspeiser T. Curious about curiosity: preliminary validity evidence for a multidimensional curiosity scale in medical students. Med Sci Educ. 2024;35(2):837–46. 40353037 10.1007/s40670-024-02242-2 PMC12058592

[20] Andrade C. The iconvenient truth about convenience and purposive samples. Indian J Psychol Med. 2021;43(1):86–8. 34349313 10.1177/0253717620977000 PMC8295573

[21] Braun V, Clarke V. Using thematic analysis in psychology. Qualitative Res Psychol. 2006;3(2):77–101.

[22] Tong A, Sainsbury P, Craig J. Consolidated criteria for reporting qualitative research (COREQ): a 32-item checklist for interviews and focus groups. Int J Qual Health Care. 2007;19(6):349–57. 17872937 10.1093/intqhc/mzm042

[23] Maslow AH. A theory of human motivation. Psychol Rev. 1943;50(4):370–96.

[24] Berlyne DE. A theory of human curiosity. Br J Psychol. 1954;45(3):180–91. 13190171 10.1111/j.2044-8295.1954.tb01243.x

[25] von Stumm S, Furnham AF. Learning approaches: associations with typical intellectual Engagement, intelligence and the big five. Pers Indiv Differ. 2012;53(5):720–3.

[26] Litman JA. Interest and deprivation factors of epistemic curiosity. Pers Indiv Differ. 2008;44(7):1585–95.

[27] Bugaj TJ, Schwarz TA, Friederich H-C, Nikendei C. The curious physician: exploring the role of curiosity in professionalism, patient care, and well-being. Ann Med. 2024;56(1):2392887. 39155851 10.1080/07853890.2024.2392887 PMC11334747

[28] Taheri F, Nasiri A. Clarifying the concept of professional curiosity in nursing: A concept analysis with walker and Avant approach. Nurs Forum. 2024;2024(1):1084372.

[29] Burgess A, van Diggele C, Roberts C, Mellis C. Facilitating small group learning in the health professions. BMC Med Educ. 2020;20(2):457. 33272270 10.1186/s12909-020-02282-3 PMC7712521

[30] Spencer J. Learning and teaching in the clinical environment. BMJ. 2003;326:591–4. 12637408 10.1136/bmj.326.7389.591 PMC1125480

[31] Roman B. Curiosity: a best practice in education. Med Educ. 2011;45(7):654–6. 21649694 10.1111/j.1365-2923.2011.04017.x

[32] Wade S, Kidd C. The role of prior knowledge and curiosity in learning. Psychon Bull Rev. 2019;26(4):1377–87. 31079309 10.3758/s13423-019-01598-6

[33] Shumylo MY. The role of elective courses in higher medical education during pre-clinical years. Sci Edu New Dimen. 2019;86(209):45–8.

[34] Lane A, McGrath J, Cleary E, Guerandel A, Malone KM. Worried, weary and worn out: mixed-method study of stress and well-being in final-year medical students. BMJ Open. 2020;10(12):e040245. 10.1136/bmjopen-2020-040245. 10.1136/bmjopen-2020-040245 PMC7733191 33303448

[35] Raupach T, Brown J, Anders S, Hasenfuss G, Harendza S. Summative assessments are more powerful drivers of student learning than resource intensive teaching formats. BMC Med. 2013;11:61. 23497243 10.1186/1741-7015-11-61 PMC3635879

[36] Abu-Zaid A. Formative assessments in medical education: a medical graduate’s perspective. Perspect Med Educ. 2013;2(5–6):358–9. 24129603 10.1007/s40037-013-0089-5 PMC3824748

[37] Epstein RM. Assessment in medical education. N Engl J Med. 2007;356(4):387–96. 17251535 10.1056/NEJMra054784

[38] Sirianansopa K. Evaluating students’ learning achievements using the formative assessment technique: a retrospective study. BMC Med Educ. 2024;24(1):1373. 39593083 10.1186/s12909-024-06347-5 PMC11590208

[39] Schattner A. An antidote to burnout? Developing broad-spectrum curiosity as a prevailing attitude. QJM. 2021;114(11):770–2. 10.1093/qjmed/hcz322 31868899

[40] Malterud K, Guassora AN, Reventlow S, Jutel A. Embracing uncertainty to advance diagnosis in general practice. Br J Gen Pract. 2017;67(659):244–5. 28546389 10.3399/bjgp17X690941 PMC5442924

[41] Marshall M. Redefining quality: valuing the role of the GP in managing uncertainty. Br J Gen Pract. 2016;66(643):e146–8. 10.3399/bjgp16X683773. 26823265 10.3399/bjgp16X683773 PMC4723214

[42] Lincoln YS, Guba EG. Fourth generation evaluation. Newbury Park, CA: Sage; 1989.

