# Supplementary material for: Exploring curiosity in undergraduate medical education: a thematic analysis
Source: BMC Med Educ. 2026 Feb 11;26:431. doi: 10.1186/s12909-026-08783-x (PMC12998176; doi:10.1186/s12909-026-08783-x)
Supplement: Supplementary file 1 — Supplementary Material 1. [file 12909_2026_8783_MOESM1_ESM.pdf]

SUPPLEMENTARY MATERIAL

COREQ (COnsolidated criteria for REporting Qualitative research) Checklist[18]

**Topic**	**Item No.**	**Guide Questions/Description**	**Reported on Page No.**
**Domain 1: Research team and reflexivity**			
Personal characteristics			
Interviewer/facilitator	1	Which author/s conducted the interview or focus group?	6
Credentials	2	What were the researcher's credentials?	22
Occupation	3	What was their occupation at the time of the study?	22
Gender	4	Was the researcher male or female?	N/A
Experience and training	5	What experience or training did the researcher have?	6
Relationship with participants			
Relationship established	6	Was a relationship established prior to study commencement?	6
Participant knowledge of the interviewer	7	What did the participants know about the researcher?	6
Interviewer characteristics	8	What characteristics were reported about the interviewer/facilitator?	6
**Domain 2: Study design**			
Theoretical framework			
Methodological orientation and theory	9	What methodological orientation was stated to underpin the study?	5
Participant selection			
Sampling	10	How were participants selected?	5
Method of approach	11	How were participants approached?	5
Sample size	12	How many participants were in the study?	6
Non-participation	13	How many people refused to participate or dropped out? Reasons?	6

Setting			
Setting of data collection	14	Where was the data collected?	6
Presence of non-participants	15	Was anyone else present besides the participants and researchers?	N/A
Description of sample	16	What are the important characteristics of the sample?	5
Data collection			
Interview guide	17	Were questions, prompts, guides provided by the authors? Was it pilot tested?	Appendix 1
Repeat interviews	18	Were repeat interviews carried out? If yes, how many?	N/A
Audio/visual recording	19	Did the research use audio or visual recording to collect the data?	6
Field notes	20	Were field notes made during and/or after the interview or focus group?	6
Duration	21	What was the duration of the interviews or focus group?	7
Data saturation	22	Was data saturation discussed?	6
Transcripts returned	23	Were transcripts returned to participants for comment and/or correction?	N/A
**Domain 3: analysis and findings**			
Data analysis			
Number of data coders	24	How many data coders coded the data?	6
Description of the coding tree	25	Did authors provide a description of the coding tree?	7
Derivation of themes	26	Were themes identified in advance or derived from the data?	6
Software	27	What software, if applicable, was used to manage the data?	6
Participant checking	28	Did participants provide feedback on the findings?	7
Reporting			
Quotations presented	29	Were participant quotations presented to illustrate the themes/findings? Was each quotation identified?	9-14

Data and findings consistent	30	Was there consistency between the data presented and the findings?	9-14
Clarity of major themes	31	Were major themes clearly presented in the findings?	9-14
Clarity of minor themes	32	Is there a description of diverse cases or discussion of minor themes?	9-14
